# Quality of Life in Patients Undergoing Surgery for Upper GI Malignancies

**DOI:** 10.3390/life13091910

**Published:** 2023-09-14

**Authors:** Grigorios Christodoulidis, Marina-Nektaria Kouliou, Konstantinos-Eleftherios Koumarelas, Dimitris Giakoustidis, Thanos Athanasiou

**Affiliations:** 1 Department of General Surgery, University Hospital of Larissa, University of Thessaly, Biopolis, 41110 Larissa, Greece; marinakouliou@gmail.com (M.-N.K.); kostaskoumarelas@gmail.com (K.-E.K.); 2 Department of Surgery, General Hospital Papageorgiou, Aristotle University of Thessaloniki, 56429 Thessaloniki, Greece; dgiakoustidis@gmail.com; 3 Department of Cardiothoracic Surgery, University Hospital of Larissa, University of Thessaly, Biopolis, 41110 Larissa, Greece; t.athanasiou@imperial.ac.uk

**Keywords:** upper GI surgery, quality of life, esophagectomy, gastrectomy, pancreaticoduodenectomy, HRQL

## Abstract

Upper gastrointestinal (GI) conditions vastly affect each individual’s physical, social, and emotional status. The decision-making process by the medical personnel about these patients is currently based on a patient’s life quality evaluation scale, HRQL scales. By utilizing HRQL scales, a better understanding of the various surgical and non-surgical treatment options, as well as their long-term consequences, can be achieved. In our study, an organ-based approach is used in an attempt to examine and characterized the effect of upper GI surgery on HRQL. Therefore, HRQL scales’ function as a prognostic tool is useful, and the need for future research, the creation of valid training programs, and modern guidelines is highlighted.

## 1. Introduction

The esophagus, stomach, and duodenum make up the upper gastrointestinal (GI) tract [1]. The main upper gastrointestinal surgeries include esophagectomy, gastrectomy, and pancreaticoduodenectomy due to malignancies. However, these operations come with considerably high rates of mortality and morbidity, affecting up to four out of ten patients [2,3]. In oncological upper GI surgeries, a complete tumor resection (R0) is performed. The digestive system is then reconstructed with a functional and appropriate anastomosis [4]. Increased intraluminal pressures in the first part of the alimentary tract, the acidic pH, as well as the gastric enzymes comprise the main physiological characteristics of the upper GI tract. A high incidence of anastomotic leak is therefore the reason for increased morbidity as well as a plethora of adverse events. It was not until 20 years ago, in 1949, when the health-related quality of life (HRQL) scale was created by Karnofsky and Burchenal, that it began to draw continuous attention using the patient’s self-evaluation as a basis [5,6]. An HRQL scale evaluates the functional performance of oncological patients. The patient-reported evaluation of HRQL also offers a way to record patients’ assessments of their symptoms, functioning, and overall well-being following surgical treatment [7,8]. Later, in 2009, the US Food and Drug Administration (FDA) advocated the creation and validation of quality-of-life indicators (patient-reported outcome measures—PROMs) that might be used for regulatory reasons [9]. Post-operatively, the GI tract structure and function are subject to physical alterations, causing eating disorders such as anorexia, decreased functional ability to eat, reflux, nausea, vomiting, and malabsorption [10,11]. Other than the nutritional repercussions, oncological patients with upper GI tract malignancies are also exposed to the risk of recurrence. Surgery, and its stress, negatively impact an individual’s HRQL. Use of a scale for self-reported HRQL during postoperative follow up is useful for identifying high-risk patients with a great possibility of decreased survival, by focusing on the postoperative functional impairment [12,13]. Based on all the currently available literature data, the goal of this study is to evaluate the effect of upper GI surgery on patient-reported HRQL. An organ-based approach is used to characterize the various effects on patients’ postoperative quality of life.

## 2. Materials and Methods

The PubMed, Cochrane Library, Medline Scopus-clinical trial register, and Web of Science databases were initially searched by the authors to retrieve studies reporting data on patient quality of life after upper GI surgery until the present day. Based on the data from the literature that is currently available, the goal of this study is to evaluate the effect of upper GI surgery on patient-reported HRQL. To characterize the various effects on patients’ postoperative quality of life, an organ-based approach was used.

## 3. Results

### 3.1. Esophagectomy and Quality of Life

Esophageal cancer is the eight deadliest cancer globally [14]. It is also the most common reason for an esophagectomy, with a 30% curative rate in early-stage cases and a 5-year survival rate of 2 to 4 out of every 10 patients. Despite a recurrence incidence of 32 to 54% in the first postoperative year and a significant risk of serious consequences for about 40% of patients, esophagectomy is still widely applied [15,16,17]. In addition to esophageal cancer, Barrett esophagus and severe achalasia also necessitate difficult and extensive surgical treatments of the esophagus [15].

During the first six months following esophagectomy, patients can develop a range of symptoms including fatigue, insomnia, oral dryness, anorexia, dysphagia, reflux, esophageal pain, diarrhea, dyspnea, cough, and diminished social function [15,18,19,20]. These symptoms can lead to a decline in health-related quality of life (HRQL) and higher death rates in oncological patients [19,21]. While QOL ratings dropped immediately after esophagectomy, an improvement back toward baseline was observed over the course of the first 100 days [22]. As for the HRQL, no connection was reported by Rutegard et al. between prolonged oncological surgical procedures, including more extensive lymphadenectomy, broader resection margins, and longer operating times, and diminished HRQL at six months after surgery [23]. Poor HRQL ratings have been triggered by complications associated with postoperative recovery, such as anastomotic leak, infections, cardiopulmonary disorders, and operative technical issues [18,21,24,25].

In order for HRQL measurements to benefit esophagectomy patients, it needs to be centered on carefully constructed ratings and assessments [26]. This will ensure that clinical practice is based on reliable and applicable guidelines. Regular monitoring and evaluation of the HRQL and implementation of symptom-specific therapies can optimize the patient’s status. Other than self-reported symptoms, reflected by routine HRQL measurement, comprise a reliable indicator (index) for rising mortality [15].

During the first semester after esophagectomy, an improved emotional performance is observed, often attributed to the patients perception that they are cancer survivors [27]. However, during the same time period, the patients become physically disabled, experiencing escalating tiredness, and breathing and digestive problems such as dyspnea and diarrhea. Twenty-one studies measuring HRQL utilizing the European Organization for Research and Treatment of Cancer (EORTC) QLQ C30 and SF-36 [28] and short-form [29] questionnaires have been analyzed in a systematic review by Scarpa et al. [27] (14 studies additionally used the disease-specific OES18 or its former version OES24). The data presented in this review affirm that esophagectomy for esophageal cancer considerably impairs both short- and long-term HRQL. Τherefore, all the research carried out in the last twenty years unanimously indicates a direct correlation between esophagectomy and an appreciable deterioration in the HRQL [30]. However, Katz et al. observed that HRQL in patients undergoing esophagectomy for malignancies improved over the course of 3 postoperative years, with their quality of life being similar to the general population [31].

The HRQL of long-term esophageal cancer survivors downscales with a substantial proportion of patients experiencing post-esophagectomy symptoms for over a year, and in rare cases, gastrointestinal issues have been reported to persist for up to 20 years [32,33,34]. Advanced age and partial treatment of underlying neoplasia exacerbate long-term progression of symptoms as well as HRQL. Jacobs et al. [35] concluded that patients experienced a permanent decline in HRQL following esophageal cancer surgery during a one-year follow-up period based on a pooled analysis of 15 original studies that assessed the quality of life among 28 HRQL variables. The impaired quality of life following esophagectomy is characterized by a rapid HRQL decline and a patient-reported HRQL decline, even in long-term, disease-free, esophageal cancer survivors. Postoperative dysphagia remains one of the principal causes of quality of life deterioration [36]. Besides the psychological advantage in precancerous instances of Barrett’s syndrome due to the perceived limited risk of carcinogenesis, esophagectomy in these cases also seems to involve a corresponding reduction in HRQL similar to that in cases of esophageal cancer [37].

Deroger et al. [38], in their study, concluded that HRQL is diminished by major postoperative complications for up to 5 years. Declines in HRQL were linked over time to variables such as surgical complications, adjuvant treatment, and possible eating disorders [24]. In the long term, only eating dysfunction seems to improve, while sleep disorders and the gastroesophageal reflux consecutively deteriorate [38]. Therefore, the reduction in quality of life after esophagectomy, due to postoperative complications, is chronic, while at the same time they only temporarily delay recovery in terms of low-grade HRQL indicators [23].

Many authors suggest that major postoperative complications may have a negative impact on patients’ long-term HRQL compared to those with minor or no postoperative complications, according to the Clavien–Dindo score. Schuring et al., in their multicenter, population-based, prospective comparative cohort study, indicated that patients who experienced major postoperative complications after esophagectomy had a greater probability of developing dyspnea, fatigue, low mood, activity intolerance, and reduced energy levels 5 years after surgery, compared to those with minor or no postoperative complications. However, only low mood, activity intolerance, and reduced energy levels were statistically significant. The same results were observed when comparing patients with minor postoperative complications to those with no postoperative complications [39].

When performing esophagectomy, surgeons often face a dilemma in choosing between the latest Minimally Invasive Esophagectomy (MIE) and conventional surgery. Selecting the most HRQL-compatible surgical method is a complex process, as six recent original research studies have shown a direct correlation between MIE and poor postoperative HRQL [40]. Specifically, overall function worsened and dyspnea, pain, fatigue, insomnia, constipation, diarrhea, cough, and speech problems persisted for six months after MIE. Speech problems and constipation persisted for 12 months and insomnia persisted for longer than 12 months [40]. Consequently, MIE and traditional surgery techniques have similar oncological outcomes. While MIE is proven to be more beneficial in the short-term, the type of surgery linked to a greater long-term advantage remains unknown [41]. At the first, sixth, and twelfth-month follow-ups after surgery, MIE significantly outperformed esophagectomy in terms of QLQC-30 and OES-18 scores [40].

Nowadays, open transthoracic esophagectomy (TTE) is considered the gold standard practice for esophagectomy [42,43]. However, open transthoracic esophagectomy (TTE) and transhiatal esophagectomy (THE) have contrasting effects in terms of postoperative quality of life. Prospective cohort studies show that those undergoing TTE experience a lower HRQL, especially during the first and second month after surgery. On the other hand, De Boers’ study presents a different perspective, supporting that although certain symptoms may linger, the HRQL is comparable to that of healthy individuals [44,45]. Furthermore, no long-term differences between the two operating technique populations were observed [46]. The HRQL decreased in both TTE and THE groups when compared to the baseline, but quickly returned to normal [46]. In addition, Ramakrishnaiah et al. showed no connection between THE and major HRQL improvement, except for the emotional function subscales [47]. For all patients with THE or TTE, HRQL helps clinicians define their survival prospects [48]. The only technique that enhances HRQL postoperatively is video-assisted thoracoscopic esophagectomy (VATS) [49]. As mentioned above, minimally invasive esophagectomy (MIE) also causes a decline in HRQL after surgery, but only during the first three months. Compared to TTE, MIE often improves overall HRQL over the 6-month follow up period after surgery [50,51].

However, when analyzing groups of patients who underwent different types of surgery such as TTE, THE, or MIE, no discernible change was found in their postoperative HRQL ratings [52,53,54,55,56]. Additionally, according to Egberts et al., the esophagogastrostomy site, as well as the type of surgery applied, had no marked impact on QoL decline [52]. Even Robotic Transhiatal Esophagectomy (Th-RAMIE), which is perioperatively advantageous, has no patient-reported superiority over THE when treating esophageal cancer [54]. Furthermore, patient comfort and quality of life remain intact regardless of the reconstruction route chosen [55]. Long et al. observed that the use of a colon conduit via the retrosternal route after esophagectomy is a safe and feasible technique that does not diminish patients’ HRQL. At the same time, long-term functional outcomes are acceptable for patients when a gastric conduit is not available [57]. Taking into account all of the above, esophagectomy itself is associated with a drastic reduction in HRQL in the 2-year period following surgery, while the surgical approach used has minimal influence on long-term symptom progression and HRQL.

The conclusion reached in this study is in complete contrast with what previous studies have indicated [58,59,60,61,62,63]. As expected, RAMIE ranks at the top of the esophagectomy techniques, leading to faster functional recovery, reduced postoperative discomfort, and improved short-term quality of life [62,63]. Robot-assisted radical transmediastinal esophagectomy, compared to TTE, was found to be related to superior long-term HRQL, as noted by Sugawara et al. [64]. Among the more common esophagectomy techniques, Ivor Lewis esophagectomy has the worst prognosis in terms of postoperative QoL. When contrasted with McKeown esophagectomy, patients undergoing Ivor Lewis experience negative impacts on physical, social, and role functioning during the first semester following a three-field lymphadenectomy and cervical esophagogastric anastomosis [58]. Additionally, in individuals with gastroesophageal junction cancer, open transthoracic Ivor Lewis esophagectomy (TTIL) causes greater discomfort and constipation than thoracoscopically assisted McKeown esophagectomy (TAMK) [60]. A greater postoperative QoL is observed in patient groups who underwent combined thoracoscopic/laparoscopic esophagectomy, compared to those who underwent Ivor Lewis and left transthoracic esophagectomy [59].

### 3.2. Gastrectomy and Quality of Life

Gastric cancer is the fifth commonest malignancy worldwide, with around 1 million new cases reported each year. It is also among those with the highest mortality rates, causing the second most deaths [65]. Nowadays, with its early diagnosis, surgery is considered the only definite treatment, with partial and total gastrectomy being the two main alternatives. Studies support that the patient’s postoperative survival rates have improved and the long-term effects on HRQL after gastrectomy are positive [66]. HRQL after major gastrectomy tends to remain intact, despite the persistent gastrointestinal symptoms such as reflux, early satiety, and episodic nausea [67]. Other defining parameters of HRQL include GI symptoms, systemic symptoms, global functioning, as well as social and psychological health [66]. Gastric cancer patients mainly suffer from alterations in the digestive system’s function, leading to metabolic disorders, including weight loss or even starvation [68].

In the majority of patients, there is a progressive HRQL enhancement in the first 12 months after surgery followed by a great attenuation in the short-term postoperative period [69,70,71,72]. The exception is for those who do not survive throughout the first postoperative year [69]. HRQL was shown to be independent of cancer stage, as Kinami et al. showed the comparability of early- and late-stage cancer HRQL [73]. Consequently, a postoperative QoL decline is inevitable. What is less clear, however, is the severity of any postoperative adverse effects on QoL, the length of impaired QoL, and the comparative effects of other surgical choices on QoL [74]. Therefore HRQL can help reduce this ambiguity.

As mentioned above, HRQL fluctuates after surgery. A systematic analysis shed more light on the precise impact of gastrectomy on HRQL. It features a 3-month decline in HRQL succeeded by evidence of long-term recovery, during the period between the 6th and the 12th month following gastrectomy [75]. Nowadays, a vast variety of newly introduced surgical techniques promises to achieve radical excision of tumors [76]. The tumor site, preoperative staging, the patient’s overall physical condition, and the surgeon’s choice based on experience are all taken into account when choosing the best course of action. Newly introduced HRQL scales give rise to a better assessment of quality of life, as they effectively reflect the patients’ self-reported evaluation of their post-operative mental and physical health, promoting the most efficient therapeutic interventions [76]. The approach of only objectively assessing the postoperative consequences should be progressively abandoned.

Modern surgery has recently introduced laparoscopic gastrectomy (LG), a less invasive laparoscopic procedure [77]. Despite LG’s increasing acceptance, open gastrectomy (OG) remains the preferred therapy when treating gastric cancer [77]. Two RCTs and a large cohort trial indicated no clinically significant difference between LG and OG patient groups in terms of postoperative functionality, symptom severity, and global health status up to one year following gastrectomy [71,78,79,80,81]. Conversely, two other studies presented a different aspect of the situation. An RCT study conducted by Kim et al. associated laparoscopic-assisted distal gastrectomy (LADG) with a 3-month postoperative boost in HRQL, advertising LADG’s superiority over DG [82]. Kobayashi et al. pointed out the validity of Kim’s study, stating that open distal gastrectomy impacts numerous categories of HRQL 12 months after surgery, while the laparoscopic method leads to improved short-term health status [83]. Single-incision distal gastrectomy (SIDG), a newly introduced technique, had similar pain scores, short-term morbidity, as well as QoL when compared to multiport laparoscopic distal gastrectomy (MLDG) according to Kang et al. based on their prospective RCT [84].

To date, several studies have been published comparing total gastrectomy (TG), distal gastrectomy (DG), partial gastrectomy (PG), and subtotal gastrectomy (STG) in terms of HRQL [85,86,87]. Physical functions, functional roles, social functions, and various other symptoms worsened more in the TG group than in the STG group, according to Kim et al. [85]. Furthermore, HRQL deteriorated three months following gastrectomy for both TG and PG, but improved six months later [86]. Using a prospective comparison analysis, Karanicolas et al. [87] found that patients undergoing PG experienced more QoL impairment than those undergoing DG or TG. In contrast, Takiguchi et al. [88] discovered in a retrospective analysis that PG offers superior HRQL compared to TG. One year postoperatively, discomfort, reflux, limited food intake, and anxiety had a greater impact on QoL in the TG group than in the DG group [89]. Short-term HRQL differences between TG and STG, on the other hand, were transient and became inconsequential a few months after surgery [90]. Additionally, an RCT comparing HRQL following D1 and D3 lymphadenectomy failed to establish a statistically significant difference between the two groups regarding the degree of gastrectomy [67]. Although proximal gastrectomy is an option, the increased incidence of bile reflux and food intolerance has led to its replacement. Irfan et al. in their single-center series indicated that, regarding bile reflux, early satiety, appetite, energy level, restrictions on physical activity, discomfort, or general discontent with their operation, there were no statistically significant differences between the groups that underwent proximal and total gastrectomy [91].

More and more anastomotic methods after gastrectomy are being studied to determine whether they have a beneficial effect on the postoperative quality of life of patients over time [76,92,93,94,95,96,97,98,99,100,101,102,103,104]. Controversial findings on pouch repair following TG have been reported. Two published in the late 1990s were smaller-scale, randomized controlled trials which demonstrated the absence of improved HRQL in the patient group who underwent a pouch reconstructive procedure [92,93]. Conversely, pouch reconstruction according to Svedlund et al. [94] and Kalmar et al. [95] offers higher postoperative HRQL and should be considered mainly for patients with well-differentiated, low-stage tumors, providing a fair likelihood of long-term survival [94,95]. Various studies have investigated Roux-en-Y (R-Y) and Billroth repair with respect to their postoperative HRQL benefits [76,96,97,98,99,100,101,102]. Patients who have R-Y reconstruction may experience improved QoL, a lower incidence of reflux esophagitis, as well as a lower incidence of epigastric pain and better HRQL findings because of the decreased angle of His in patients following RY compared with Billroth repair [76,96,105]. Smolskas et al. [100], on the other hand, observed that patients who had Billroth I reconstruction had the highest QoL scores. The majority of investigations, however, indicated that there were no differences in QoL amongst the reconstruction methods utilized [97,98,101]. After a fully laparoscopic TG, isoperistaltic anastomosis (IJOM) has been linked to greater HRQL scores than R-Y anastomosis [103]. Ikeda et al. compared the use of the esophagogastrostomy method (PGEG) and double tract method (PGDT) after proximal gastrectomy. Although PGEG contributed to better weight loss, PGDT had substantially improved constipation and dumping SS scores (*p* < 0.05). PGDT had significantly better diarrhea SS ratings, less discontent with symptoms, and better dissatisfaction with daily life SS scores (*p* < 0.05) [106].

## 4. Discussion

It is essential that the long-term effects of therapies are more clearly understood, surgical and medical therapy options should be better compared, and indications should be provided when there are no other objective variables [104,105,106,107]. HRQL data are an essential tool that contributes to all these aspects. The influence of the disease and its therapies on patients’ physical, social, and emotional health, measured through HRQL, may be accurately assessed in patients with upper gastroenterological problems, which is useful for therapeutic decision making. Three key domains—general function, well-being perception, and subjective symptoms—are used to assess the HRQL of patients with upper gastrointestinal illnesses [107,108]. Patients’ viewpoint on the outcome and impact of the therapy as well as the experiences of patients suffering from gastric and esophageal cancer, which are characterized by a poor prognosis, are imprinted in HRQL [76,109]. HRQL data can also be used to calculate quality-adjusted life years (QALY), a crucial tool for health economic assessments [110]. Therefore, HRQL evaluation at baseline, pre-treatment, and post-treatment is an important tool in decision making. Younger patients are more likely to accept a major surgery to increase their survival, without taking into account the impact on their QoL. However, postoperative complications and morbidities and their effect on patients’ HRQoL play an important role in decision making for elderly patients, as undergoing these therapies may lead to regret of their choices [111]. The present research has a significant limitation, as the majority of the evidence supporting the predictive significance of HRQL in esophagogastric cancer comes from RCTs and population-based studies, with the former exceeding the latter [112]. These trials have rigorous inclusion criteria, so the typical trial patient only represents a fraction of the target group, and systematic bias is quite likely given that the real-world cancer population is not abundantly represented in RCTs [113].

The evaluation of HRQL is of utmost importance, particularly for patients scheduled for upper GI surgery. By allowing patients to report on a variety of symptoms, such as psychological side effects from medications and illness, through a wide range of generic instruments and disease-specific PROMs, this evaluation becomes possible [114]. The 36-Item Short Form Survey (SF-36) is a widely accepted and utilized general instrument [115]. In addition, the Patient Reported Outcomes Measurement Information System (PROMIS) questionnaires are a well-validated and practical method for evaluating HRQL in a range of patient groups as they are intended to measure physical, mental, and social health internationally [116]. To gain information about the symptoms experienced by patients with specific diseases, approaches to assess the HRQL in patients undergoing upper GI surgeries can be utilized. The ratings can also be applied to comparative efficacy studies of various therapeutic modalities and perhaps even to prognostication in malignant illness [114]. The European Organization for Research and Treatment of Cancer Quality of Life Questionnaire-Core 30 questionnaire (EORTC QLQ-C30) and the Functional Assessment of Cancer Therapy-General (FACT-G) [117,118] are two broadly applied PROMs in oncological patients. The most popular HRQL tool in esophagogastric cancer RCTs is the validated EORTC QLQ-C30 questionnaire [119], commonly regarded as the “standard” HRQL scale.

Psychological disorders, such as anxiety and depression, are prevalent in this patient population. Therefore, follow-up care is essential to manage the curative therapy’s side effects and provide psychological assessment and support to patients recovering from cancer, as well as to detect early recurrence. However, high-intensity follow-up and frequent hospital visits significantly influence patients’ lives. Wullaert et al. conducted a meta-analysis and found that low-intensity follow-up has no negative effects on patients’ HRQL [120].

General HRQL instruments are not specific to the symptoms experienced by cancer patients, particularly those with upper gastrointestinal cancer, who commonly experience symptoms related to eating and drinking. To address this issue, a cancer-specific questionnaire with a site-specific module is required [121]. Multiple studies have shown a decline in overall HRQL following therapy while site-specific disorders, such as dysphagia, are corrected, underscoring the need for site-specific modules [121,122].

Due to a lack of HRQL-specific protocols, materials, training, and education, HRQL studies in the existing literature present inconsistent standards for HRQL data collection. Compared to other studies that report their HRQL findings together with the key clinical outcomes, RCTs that focused on HRQL as their primary endpoint were more likely to be of higher quality [119,123,124]. However, HRQL trials might be biased by these discrepancies [125]. To ensure high-quality data collection, future research should develop HRQL guidelines and training programs.

Nowadays, the operative mortality and morbidity of major surgeries, such as esophagectomy, have been reduced to tolerable levels. Nonetheless, patients’ expectations primarily focus on survival, rather than taking other HRQL factors into account. Medical personnel must continue to strive for enhancing these patients’ postoperative quality of life [126]. Although there are many obstacles to reliable optimization of postoperative quality of life, a subgroup of patients (those with extensive locally advanced disease that is associated with a poor prognosis or high perioperative risk patients) may benefit the most from nonsurgical treatment [126]. The HRQOL measure appears to be more sensitive to surgical and non-surgical treatment, and also seems to predict survival better than clinician-derived performance status [127]. HRQOL evaluation has the potential to be a significant tool in assisting collaborative decision making, directing treatment planning and tracking treatment success [127].

## 5. Conclusions

Pre-upper GI surgery estimations and evaluations for every individual’s HRQL can vastly benefit the decision-makssing process when it comes to each patient’s therapeutic plan. When it comes to an already deteriorated HRQL, Th-RAMIE, VATS, and MIE induce a smaller impact on HRQL scores after esophagectomy, and LADG as well as SIDG are the preferred choices when TG can be avoided. Therefore, the need for greater knowledge in this field gives rise to the performance of new high-quality studies aimed at the development and introduction of HRQL training programs and recommendations and their application in daily clinical practice.

## Data Availability

The data presented in this study are available on request from the corresponding author.
